# Supplementary figures and images for: Kiaa1024L/Minar2 is essential for hearing by regulating cholesterol distribution in hair bundles
Source: eLife. 2022 Nov 1;11:e80865. doi: 10.7554/eLife.80865 (PMC9714970; doi:10.7554/eLife.80865)

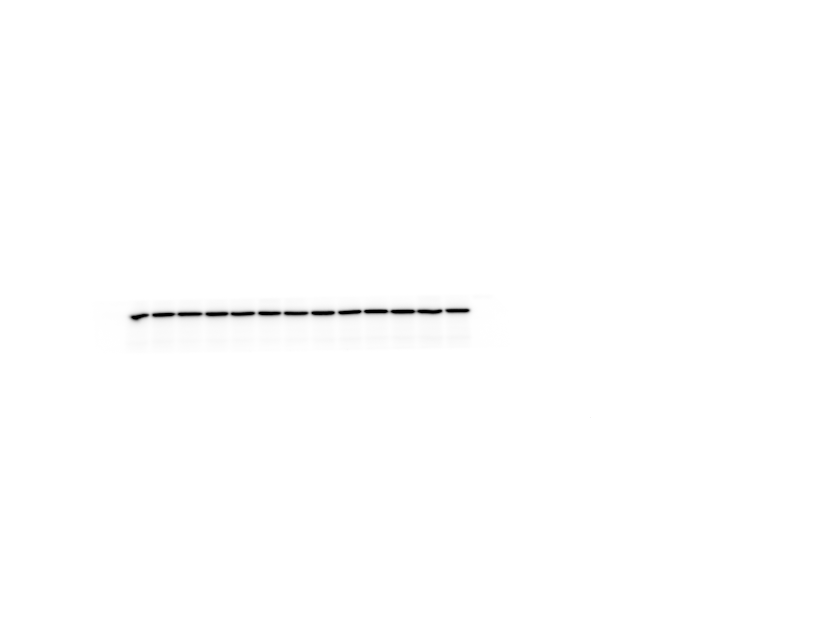

Supplement: Figure 4—source data 1. [file elife-80865-fig4-data1.zip › Figure 4-figure supplement 1C Actin raw.png]

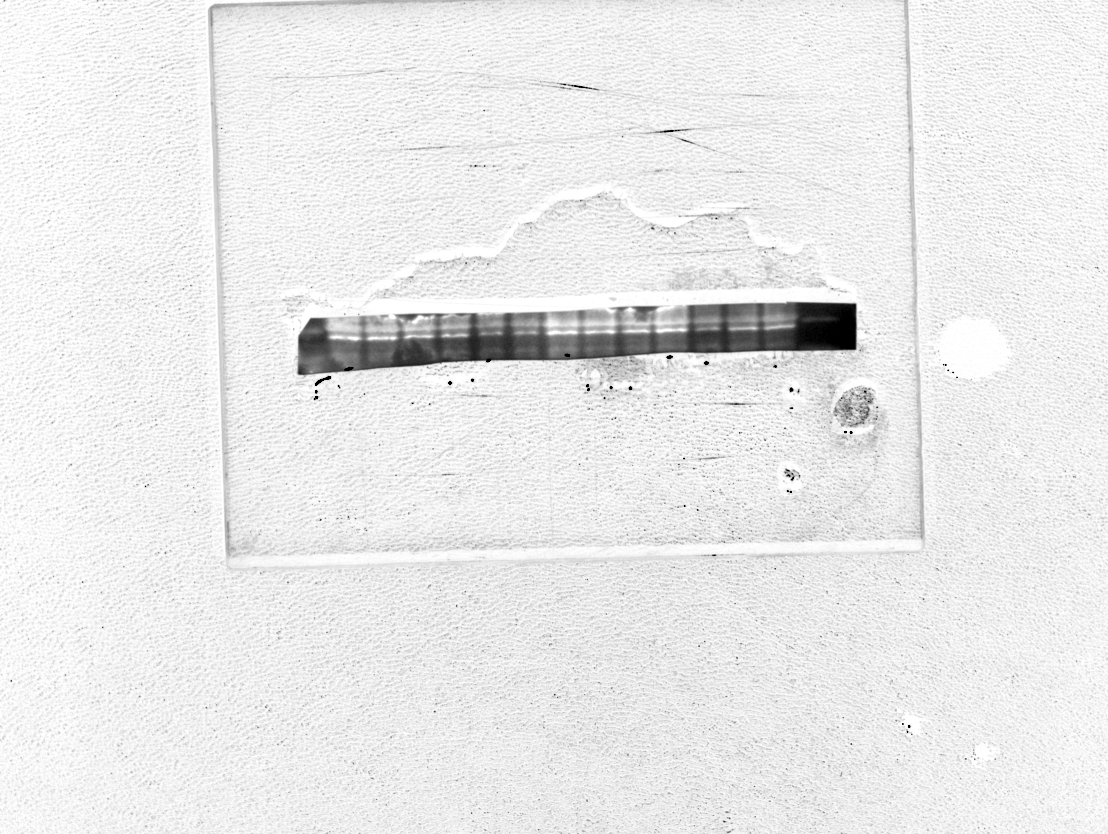

Supplement: Figure 4—source data 1. [file elife-80865-fig4-data1.zip › Figure 4-figure supplement 1C D4H-mCherry raw.png]

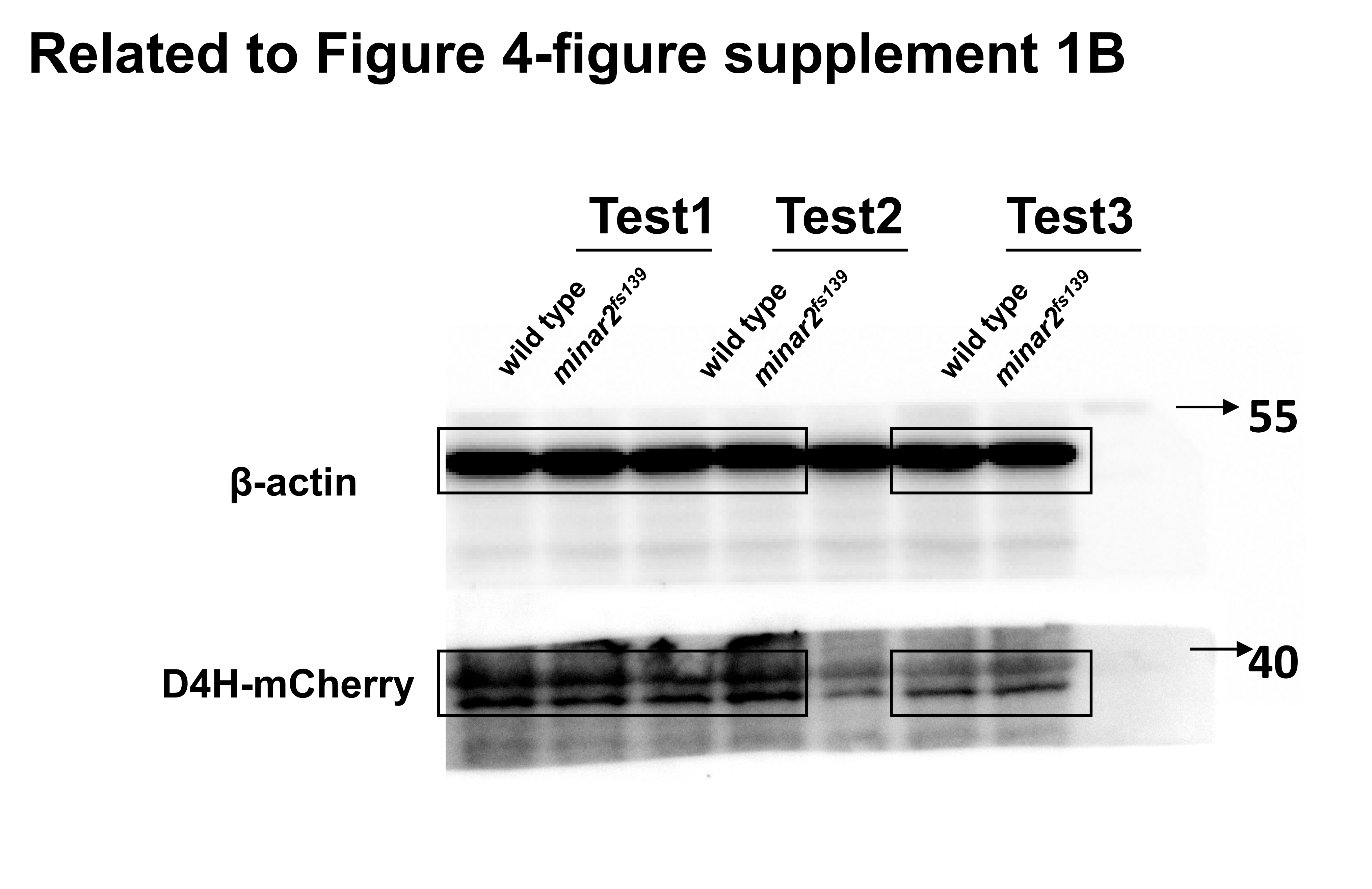

Supplement: Figure 4—source data 1. [file elife-80865-fig4-data1.zip › Figure 4-figure supplement 1C.TIFF]

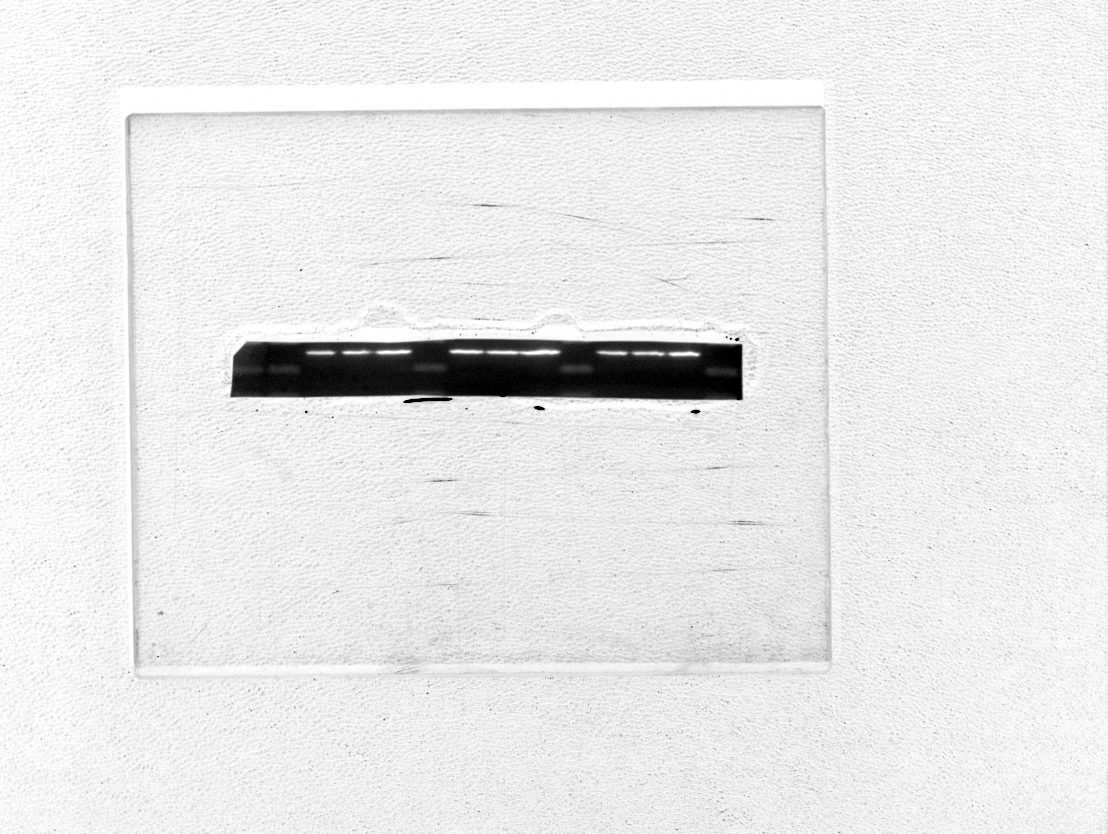

Supplement: Figure 7—source data 1. [file elife-80865-fig7-data1.zip › Figure 7C GAPDH raw.png]

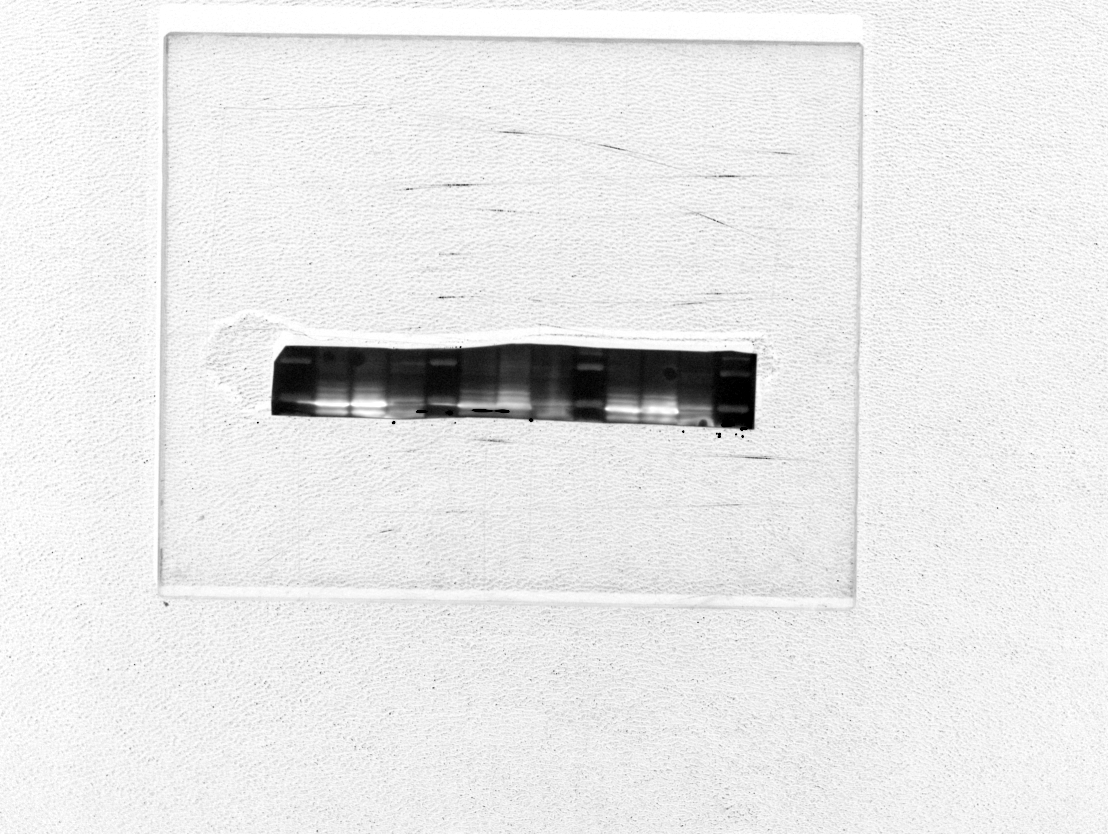

Supplement: Figure 7—source data 1. [file elife-80865-fig7-data1.zip › Figure 7C GFP raw.png]

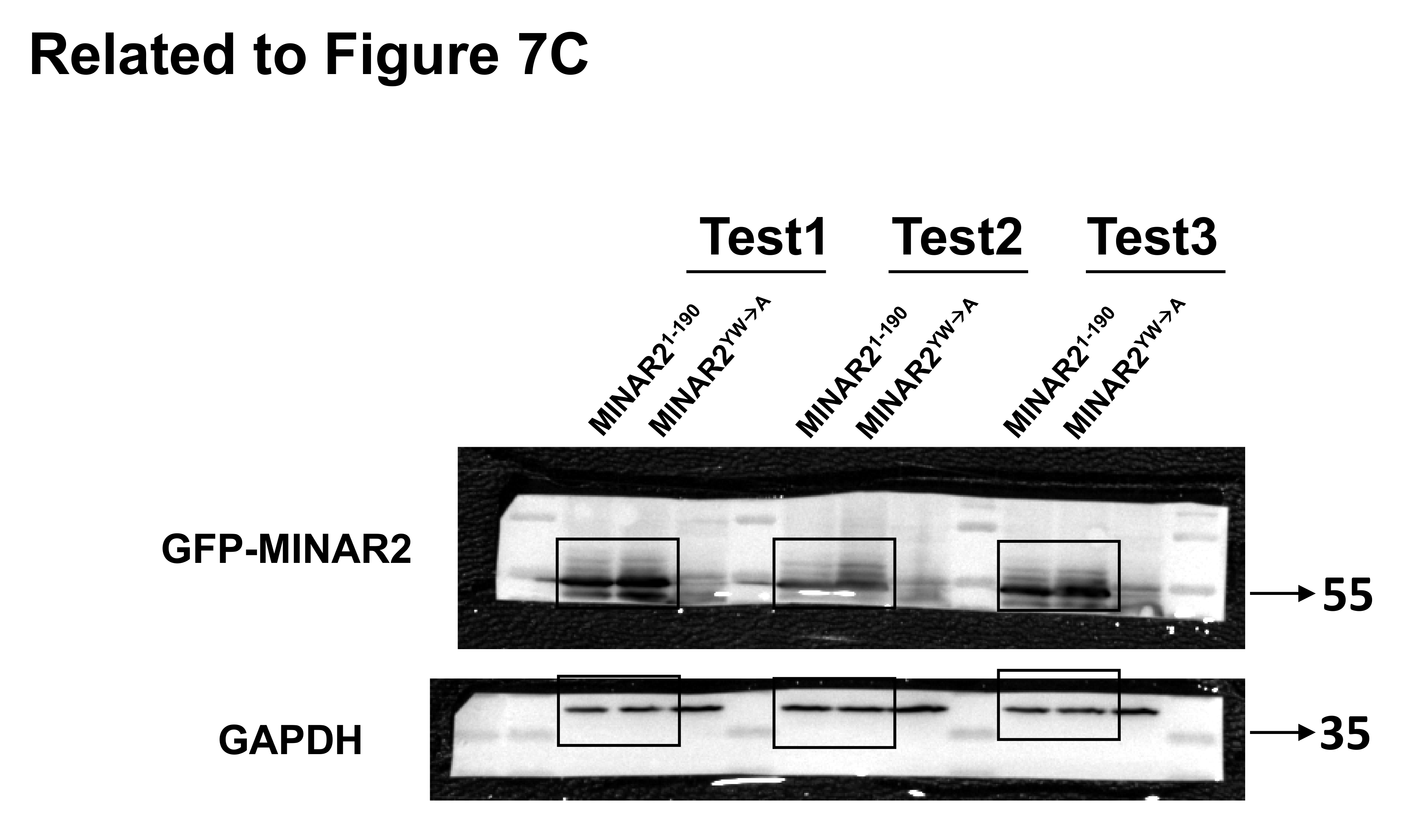

Supplement: Figure 7—source data 1. [file elife-80865-fig7-data1.zip › Figure 7C.TIFF]
